# Mapping Novel Frataxin Mitochondrial Networks Through Protein- Protein Interactions

**DOI:** 10.21203/rs.3.rs-4259413/v1

**Published:** 2024-04-26

**Authors:** Etienne Gnimpieba, D M Diing, Jared Ailts, Anja Cucak, Olaksandr Gakh, Grazia Isaya, Seasson Vitiello, Shirley Wang, Paul Pierce, Alec Cooper, Kyle Roux, Lynette K. Rogers, Peter F. Vitiello

**Affiliations:** University of South Dakota; University of South Dakota; University of South Dakota Sanford School of Medicine; Sanford Research; Mayo Clinic; Mayo Clinic; University of Oklahoma; University of Oklahoma Health Sciences Center; University of Oklahoma Health Sciences Center; University of Oklahoma Health Sciences Center; Sanford Research; University of Oklahoma Health Sciences Center; University of Oklahoma Health Sciences Center

**Keywords:** frataxin, Friedreich’s Ataxia, BioID2, protein landscape, mitochondria, peroxiredoxin

## Abstract

Friedreich’s Ataxia (FRDA) is a neuromuscular degenerative disorder caused by trinucleotide expansions in the first intron of the frataxin (FXN) gene, resulting in insufficient levels of functional FNX protein. Deficits in FXN involve mitochondrial disruptions including iron-sulfur cluster synthesis and impaired energetics. These studies were to identify unique protein-protein interactions with FXN to better understand its function and design therapeutics. Two complementary approaches were employed, BioID and Co-IP, to identify protein interactions with FXN at the direct binding, indirect binding, and non-proximal levels. Forty-one novel protein interactions were identified by BioID and IP techniques. The FXN protein landscape was further analyzed incorporating both interaction type and functional pathways using a maximum path of 6 proteins with a potential direct interaction between FXN and NFS1. Probing the intersection between FXN-protein landscape and biological pathways associated with FRDA, we identified 41 proteins of interest. Peroxiredoxin 3 (Prdx3) was chosen for further analysis because of its role in mitochondrial oxidative injury. Our data has demonstrated the strengths of employing complementary methods to identify a unique interactome for FXN. Our data provides new insights into FXN function and regulation, a potential direct interaction between FXN and NFS1, and pathway interactions between FXN and Prdx3.

## INTRODUCTION

Friedreich’s Ataxia (FRDA) is a neuromuscular degenerative disorder characterized by fatigue, cerebellar ataxia, and hypertrophic cardiomyopathy^1^. Most patients carry homozygous GAA trinucleotide expansions in the first intron of the frataxin-encoding gene (FXN), resulting in condensation of chromatin and impaired FXN transcription. FXN protein localizes to the mitochondrial matrix and is associated with the inner mitochondrial membrane. Insufficient levels of FXN protein causes several mitochondrial deficiencies, including reduced iron-sulfur cluster synthesis, impaired energetics and dynamics, and accumulation of labile iron and reduced oxygen species^2^. Although these mitochondrial deficiencies have been associated with FRDA pathogenesis, there are still mechanistic gaps as to how loss of FXN expression results in mitochondrial dysfunction.

Biochemical studies with human FXN and highly conserved homologues have shown that FXN delivers ferrous iron to the iron-sulfur cluster (ISC) synthesis complex and forms physical contacts with core proteins NFS1, ISCU and ISD11^3^. As reviewed by Isaya^4^, ISC formation is widely accepted as the primary function of FXN since reduced levels of FXN in FRDA samples and mouse models have resulted in reduced activities of iron-sulfur dependent enzymes including complexes I-III of the respiratory chain and mitochondrial aconitase^5–7^. Another consequence of impaired ISC biosynthesis is the accumulation of labile iron in mitochondria which may also further impair heme synthesis during FXN deficiency^8–10^. Although there are conflicting reports of reproducibility and functional consequences of iron accumulation in FRDA^11–14^, any increase in redox-active iron in combination with dysregulated coupling efficiency due to reduced respiratory complex activities can impair energy metabolism and augment localized formation of reduced oxygen species^15^. Such mitochondrial oxidative perturbations and saturation and/or loss of antioxidant activities have been associated with FRDA progression^16–19^.

Current FRDA therapeutic strategies based on correcting the aforementioned mitochondrial pathologies during FXN deficiency include delivery of mitochondrial-targeted iron chelators and redox-active compounds^20–22^, as well as chemical induction of cellular antioxidants and mitochondrial biogenesis via nuclear factor erythroid 2-related factor 2 (Nrf2)^23–26^. These strategies aim to alleviate functional consequences of reduced FXN levels but do not directly address specific molecular targets because our current knowledge of the FXN protein network is largely limited to ISC biosynthesis. The current investigation sought to better define the FXN protein network in human cells using two complementary approaches: proximity-based labeling (BioID) and endogenous co-immunoprecipitation (co-IP). The discovery of these new FXN protein interactions provides opportunities to understand essential FXN-dependent functions as well as identify new molecular and functional therapeutic targets in FRDA.

## RESULTS

### Expression of FXN and MTS-Cox8a transgenes in A549 cells.

**Figure 1:**
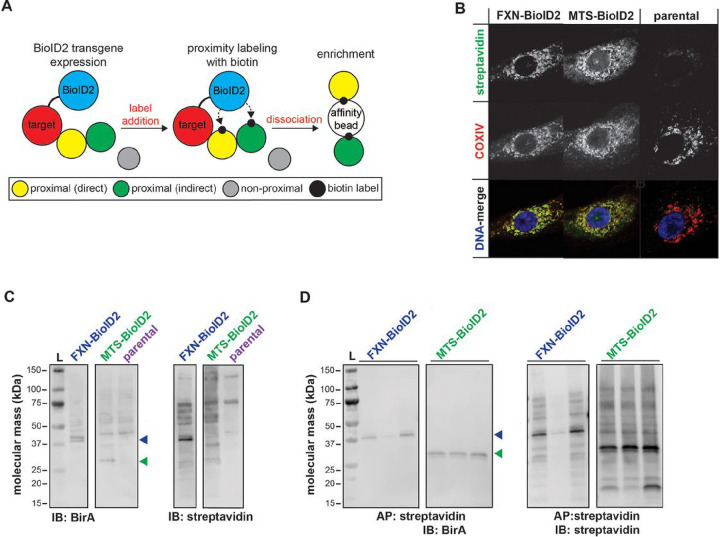
Mitochondrial localization and biotinyl ligase activity of BioID2 transgenes. (A) BioID2 was cloned in-frame with carboxy terminus of human FXN, Prdx3, and the MTS of COX8a for detection and enrichment of proximal interacting proteins. (B) Immunofluorescent labeling of parental and stably transfected A549 cells expressing BioID2 transgenes that were pulsed with 50 μM biotin for streptavidin (green) and COXIV (red) to visualize transgene expression, subcellular localization, and biotinyl ligase activity. Dapi was used as a nuclear counterstain (blue in DNA-merge). SDS-PAGE/immunoblots of (C) whole cell lysates or (D) streptavidin affinity purified A549 BioID2 cell lysates with anti-BirA antibody or streptavidin for transgene detection and protein biotinylation. Colored arrowheads indicate detection of predicted-sized transgenes.

Mitochondrial localization of transgene biotinyl ligase activity (BirA*) was confirmed by co-localization of streptavidin and cytochrome oxidase subunit IV (COXIV) in FXN-BioID2 and MTS-BioID2 cell lines (Fig. 1A and 1B). Cells were pulsed with biotin and biontinylated proteins were affinity purified with streptavidin-conjugated sepharose beads. SDS-PAGE/immunoblot of eluted fractions confirmed unique biotinylation patterns between FXN-BioID2 and MTS-BioID2 controls (Fig. 1C). Samples were then subjected to LC-MS/MS analysis for peptide identification (Supp File 1).

### Identification of FXN protein networks by co-IP.

**Figure 2:**
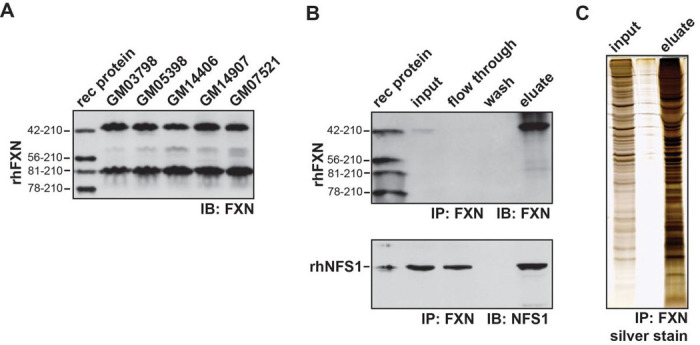
Immunoprecipitation of endogenous FXN from human lymphoblasts. (A) SDS-PAGE/immunoblot of recombinant human FXN (rhFXN) and human lymphoblast lysates with anti-FXN antibody. (B) SDS-PAGE/immunoblots of rhFXN, recombinant human NFS1 (rhNFS1) and anti-FXN immunopurified samples from pooled fractionated human lymphoblast lysates probed with FXN and NFS1 antibodies. (C) Silver staining of lysate input and anti-FXN immunopurified eluate from pooled fractionated human lymphoblast lysates.

Two functional isoforms of FXN have been identified,FXN^42−210^, a full-length form which donates either Fe^2+^ or Fe^3+^ for ISC assembly and FXN^81−210^,a truncatedform that is thought to only donate Fe^2+^.Loss of FXN^42−210^ functionality may be critical for disease pathogenesis since expression of this isoform is lost to a greater extent compared to FXN^81−210^ in FRDA patient lymphoblasts and cerebellum^27^. Three human lymphoblast lines from healthy controls (GM03798, GM05398, GM14907) were selected based on higher expression of FXN^42−210^ (Fig. 2A)^27^. Cell lysates were pooled and other shorter FXN isoforms were excluded by size exclusion chromatography (Fig. S1). Co-IP with FXN protein was used as a complementary proteomics approach to identify FXN protein networks. Furthermore, FXN^42−210^-positive fractions were selected for co-IP based on purity and co-fractionation with NFS1 (Fig. S1). Successful FXN co-IP was confirmed by NFS1 detection in eluate (Fig. 2B). Eluate was subsequently separated by SDS-PAGE and bands were excised for tandem mass spectrometry (Fig. 2C).

#### Construction of the FXN-protein interaction landscape.

**Figure 3:**
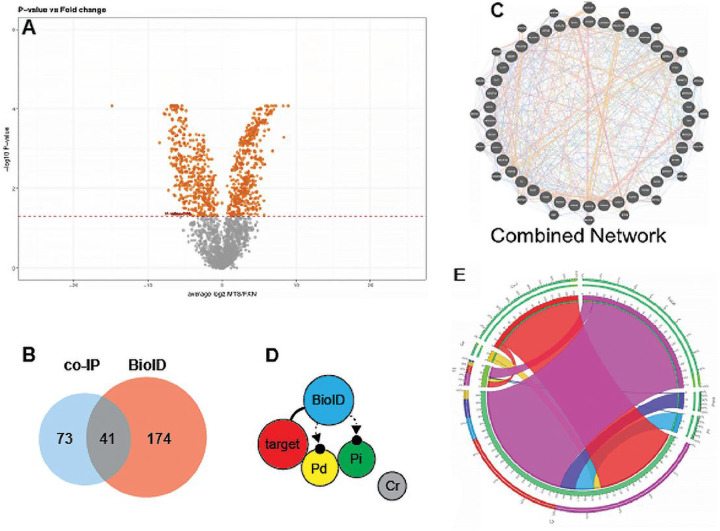
Discovery of FXN-protein interaction landscape. An interactome of FXN with others localized proteins of mitochondrion forming a network of direct and indirect protein-protein reaction was constructed. A) volcano plot shows selected protein at 0.05 cut-off. B) BioID detects protein affinity at three levels: direct binding, indirect binding, and non-proximal. 196 proteins using BioID, 117 proteins using co-IP, and 41 proteins identified with both methods were detected with high affinity for FXN. C) using the PSICQUIC collection interactome databases, we discovered 13,500 interactions (co-expression, co-localization, physical interaction) connecting our 41 proteins. D) using the interaction collection of the 41 proteins, UniProt ID or gene symbol search queries were used to generate a path from FXN to each protein. E) the maximum path from FXN to any target protein in the landscape is 6 when using either UniProt ID or gene symbol.

Following data preprocessing, manual curation using reference databases confirmed that 93.95% and 97.44% of proteins identified using BioID and co-IP respectively were localized to mitochondria (Table 1). In total, we identified 196 and 117 proteins with high affinity for FXN by BioID and co-IP respectively (Supp File 1), as well as an interaction collection of 41 proteins identified using both methods (Table 2, Fig 3A). Specificity achieved by integrating data validated using both methods allowed us to build and curate an FXN-protein interaction landscape, relying on the hypothesis that interaction confidence directly correlates with protein affinity strength. This hypothesis is supported by the BioID mechanism of action which detects protein affinity at three levels: direct binding, indirect binding, and non-proximal (Fig 3B).

After integrating 15 interactome databases from the PSICQUIC collection, we discovered 13,500 interactions (co-expression, co-localization, physical interaction) connecting our 41 proteins (Fig 3C, Supp File 2). A visual example of network density and complexity was generated based on the highly curated interactome GENEMANIA (Fig. 3D, Supp File 3). Despite the density of our network, close relationships between our proteins of interest and FXN across various interaction types at both protein and gene network levels were demonstrated. Using our interaction collection of 41 proteins, UniProt ID or gene symbol search queries were used to generate a path from FXN to each protein (Fig. 3D). The maximum path from FXN to any target protein in the landscape is 6 when using either UniProt ID or gene symbol (e.g. FXN > GRPEL1 > MRPL9 > MRPL10 > MCCC2 > PCCB > THEM4) (Fig 3E).

#### Intersection of FXN-protein interaction landscape with biological pathways implicated in Friedreich’s Ataxia.

**Figure 4:**
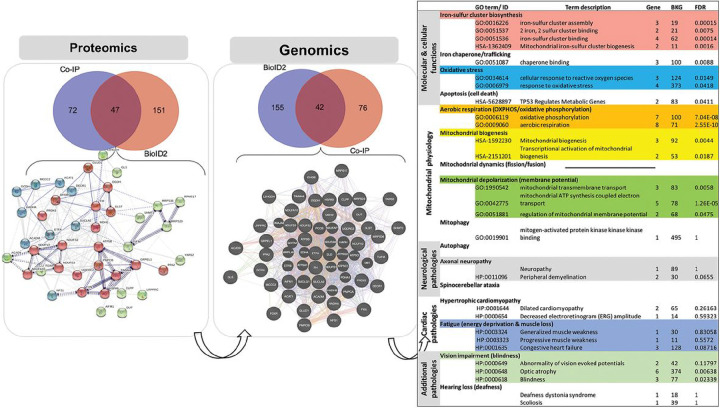
Biosystem & network analyses. Integration analysis was performed using the FXN-protein interaction landscape and the intersection with biological pathways implicated in Friedreich’s Ataxia. Using the 41 identified protein interactions and 20 selected biosystems with relevance to FXN function, mitochondrial biology and FRDA pathology, and discovery workflow identified 35 new direct and 18 indirect interactions.

Protein and gene interaction networks were generated based on our interaction collection of 41 proteins to integrate protein-protein interactions and gene regulatory networks with biological functions (Fig. 4). The systems biology landscape focused on 20 selected biosystems of interest with relevance to FXN function, mitochondrial biology, and FRDA pathogenesis. The discovery workflow determined 35 new direct and 18 indirect interactions across protein and gene networks categorized by interaction type (physical interaction, shared protein domain, etc.) (Tables 1 and 2). Our interaction network also increased connectivity between selected molecular biosystems, shortening pathway distance from FXN (Fig. 4). Box 1 describes a simple toy example to demonstrate how the level of interaction in FXN-protein landscape can inform our understanding of molecular paradigms influencing biosystems. Existing interactome databases (GENEMANIA, STRINGdb) support FXN-NFS1 interactions but this is dependent on ISD11 (also known as LYRM4) as an intermediate ^28^. However, ISD11 was not detected by either BioID or co-IP, reinforcing our landscape construction with novel direct affinity between FXN and NFS1. This new discovery empowers our understanding of ISC assembly (gene ontology 0016226), which has previously been associated with FXN function and FRDA pathogenesis.

### Network-pathways integration reveals interactions between FXN and the ISC synthesis complex and peroxiredoxin 3 (Prdx3).

**Figure 5:**
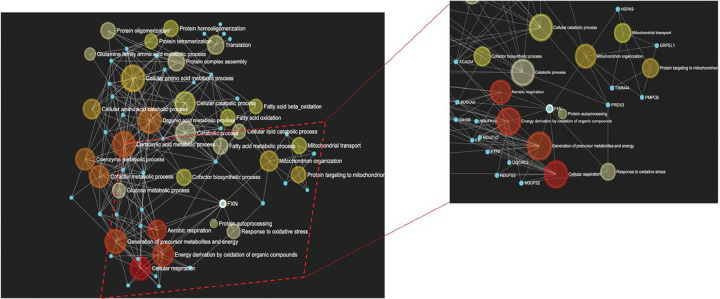
Interactions between FXN and the ISC synthesis complex and peroxiredoxin 3 (Prdx3) by network-pathways integration. A cord diagram illustrates the 41 proteins in the FXN protein landscape integrating both interaction type with functional pathways. Prdx3 was identified as a target of interest.

We constructed a cord diagram mapping our 41 proteins in the FXN protein landscape integrating both interaction type (e.g. physical, co-expression, etc) with functional pathways (Fig. 5). Using this network-pathways integration supporting identification of shared interactions to pathways relevant to FXN and FRDA pathogenesis, we identified Prdx3 as a target of interest. Prdx3 is the primary oxidoreductase in mitochondria responsible for metabolizing H_2_O_2_^29^. Therefore, Prdx3 could play a pivotal role in both ISC synthesis and FRDA pathogenesis by detoxifying localized production of H_2_O_2_ as a byproduct of Fenton chemistry occurring from reactivity of labile iron. FXN-Prdx3 pathway interactions depicted in Fig. 5 include response to oxidative stress and mitochondrial organization. FXN, and Prdx3 are involved in biological processes/pathways function of mitochondria organization and response to oxidative stress. They are also involved with several transporters or importer proteins/ genes that seem to play essential roles in the mechanism of FXN and Prdx3 functions (GRPEL1, HSPA9, TIMME4, PMBC, etc.), warranting possible participation in the same pathways as Prdx3 and FXN. Here, PMBC and GRPEL1 in the pathway act as modifier and transporter proteins. To confirm these findings, a reverse BioID screen was used to probe for Prdx3 interactions with ISC complex proteins. Expression of Prdx3-BirA* (biotinylated ligase A, R118G mutation) increased detection of biotinylated proteins which were localized to mitochondria of stably transfected A549 cells (Fig. 1B–D, Fig S2). Although FXN was not detected by mass spectrometry, prominent ISC synthesis complex proteins ISCU and NFS1 were identified out of 41 unique peptides (Supp File 1).

### FRDA and Prdx3 levels using mouse and cell models.

**Figure 6:**
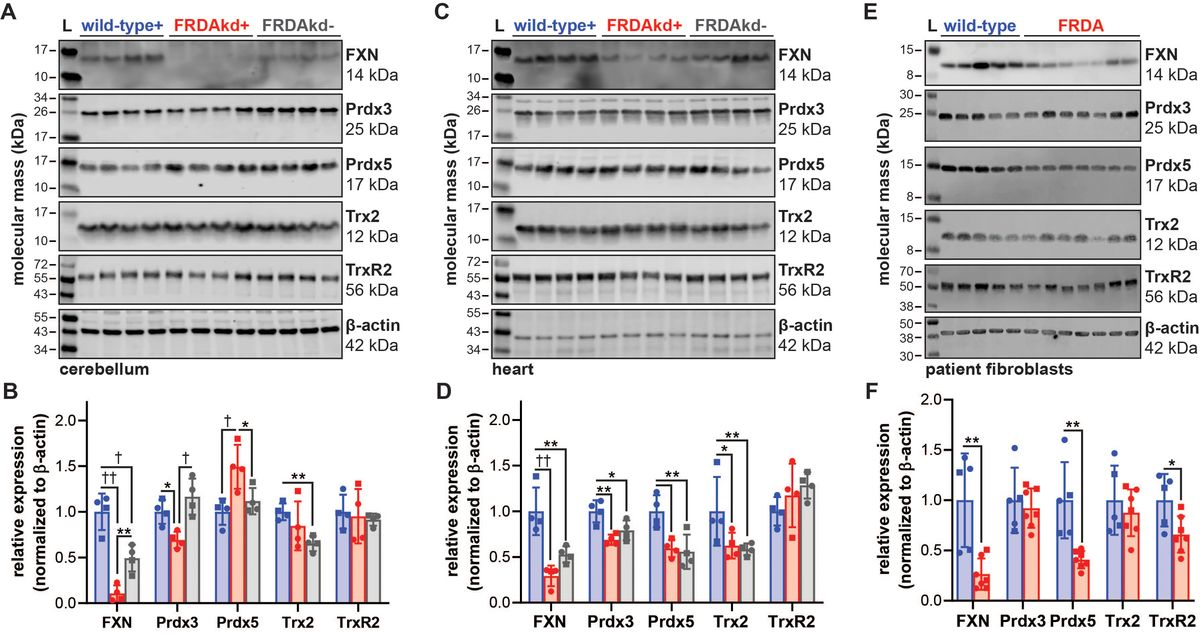
Expression of mitochondrial thioredoxin family enzymes in FRDA mouse and cell models. SDS-PAGE/immunoblot of (A) cerebellum and (C) heart lysates from wild-type and FRDAkd mice treated with (+) or without (−) doxycycline water for 18 weeks. Densitometry data from (B) cerebellum and (D) heart are expressed as mean ± standard deviation depicting 4 female (circle) and male (square) mouse subjects analyzed by one-way ANOVA. (E) SDS-PAGE/immunoblot and densitometry of lysates from dermal fibroblasts isolated from control and FRDA patients. (F) Densitometry data from patient fibroblasts expressed as mean ± standard deviation of 5–7 cell lines depicting female (circle) and male (square) subjects analyzed by a student’s t test. Lysates were probed with antibodies against FXN, Prdx3, Prdx5, Trx2, and TrxR2 with β-actin as a loading control. Statistical significance is defined as *p<0.05, **p<0.01, ^†^p<0.001 and ^††^p<0.0001.

Mouse and cell models of FRDA were used to investigate differential expression of Prdx3 or related mitochondrial thioredoxin-dependent enzymes including peroxiredoxin 5 (Prdx5), thioredoxin 2 (Trx2), and thioredoxin reductase 2 (TrxR2), between disease and control states. FRDA knockdown (FRDAkd) mice have systemic doxycycline-inducible knockdown of FXN recapitulating multiple phenotypes observed in patients ^30^. We were able to reproduce similar phenotypes in FRDAkd mice (Fig. S5A) after 18 weeks of continuous access to doxycycline in drinking water. FRDAkd mice treated with doxycycline exhibited ataxic behaviors including increased hindlimb clasping, poor coordination when walking on a ledge, and decreased grip strength, even though gait was not changed (Fig. S3B-F). Importantly, FRDAkd mice that did not receive doxycycline did not display any behaviors different from that of wild-type controls, indicating that behavioral changes are FXN-dependent. However, FXN expression was decreased compared to wild-type in transgenic FRDAkd mice independent of doxycycline (Fig. 6A–D). Therefore, there may be FXN dose-dependent effects between wild-type and transgenic mice prior to doxycycline administration which was not previously reported ^30^. Decreased Prdx3 and increased Prdx5 were detected in cerebellum of FRDAkd mice receiving doxycycline (Fig. 6A and B). This was not recapitulated in cardiac tissue (Fig. 6C and D), suggesting tissue-specific changes despite systemic FXN knockdown. However, FXN-dependent changes in expression of mitochondrial thioredoxin enzymes in cardiac tissue could be masked by decreased FXN in FRDAkd mice in absence of doxycycline. A panel of patient-derived dermal fibroblasts were used as a second model to investigate FXN-dependent changes. FRDA fibroblasts expressed 26.5% FXN compared to controls and had decreased expression of Prdx5 and TrxR2 (Fig. 6E and F). Loss of Prdx5 expression in FRDA lymphoblasts was previously reported by Hayashi and Cortopassi while performing a targeted screen of oxidative stress genes ^19^. There were no changes in expression of the mitochondrial chaperone HSP60 in either FRDAkd samples or lymphoblasts, suggesting that FXN-dependent changes in mitochondrial antioxidant protein expression was not due to altered mitochondrial content (data not shown).

### Prdx3 redox status in FRDA mice and cell models.

**Figure 7:**
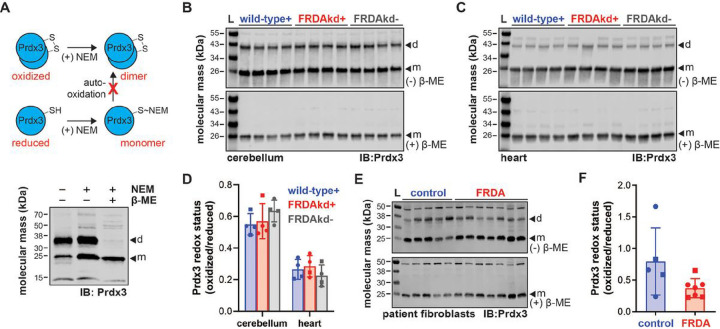
Prdx3 redox status in FRDA mouse and cell models. (A) Schematic of Prdx3 redox labeling by NEM alkylation of free thiols to inhibit artificial auto-oxidation and dimerization during sample processing. SDS-PAGE/immunoblot of lysates from control patient dermal fibroblasts subjected to NEM alkylation and/or β-mercaptoethanol (β-ME) supplementation in Laemmli buffer then probed with antibodies against Prdx3 (d=dimer, m=monomer). SDS-PAGE/immunoblot of NEM-treated (B) cerebellum and (C) heart lysates from wild-type and FRDAkd mice treated with (+) or without (−) doxycycline water for 18 weeks under non-reducing or reducing conditions (−/+ β-ME in Laemmli buffer) and probed with antibodies against Prdx3 (d=dimer, m=monomer). (D) Densitometry data from cerebellum and heart are expressed as mean ± standard deviation depicting 4 female (circle) and male (square) mouse subjects analyzed by one-way ANOVA. (E) SDS-PAGE/immunoblot of NEM-treated lysates from FRDA patient dermal fibroblasts with control age/sex-matched subjects under non-reducing or reducing conditions (−/+ β-ME in Laemmli buffer) and probed with antibodies against Prdx3 (d=dimer, m=monomer). (F) Densitometry data from patient fibroblasts are expressed as mean ± standard deviation of 5–7 cell lines analyzed by a student’s t test.

Redox cycling of Prdx3 is critical in dictating oxidoreductase activity. As depicted by the schematic in Fig. 7A top, Prdx3 redox state was determined across similar biological samples by differentiating Prdx3 oxidized dimers from reduced monomers through differential n-ethylmaleimide (NEM) alkylation of free thiols. Preservation of Prdx3 dimers under non-reducing conditions was confirmed by loss of signal following reduction with β-mercaptoethanol (Fig. 7A, bottom). The reduced monomeric form of Prdx3 was more prevalent than oxidized dimers in mouse cerebellum and heart and this was not affected by FXN knockdown (Fig. 7B, C and D). Again, specificity of oxidized Prdx3 dimers were confirmed by reducing lysates with β-mercaptoethanol. Similarly, no significant changes in Prdx3 redox state were detected in patient-derived fibroblasts (Fig. 7E and F).

## DISCUSSION

Decreased or defective FXN expression and its role in the development of FRDA is poorly understood. Little is known about FXN interactions with other proteins, specifically in assembly of ISC, and the function cluster synthesis and iron metabolism. Previous metabolomic-based studies have identified defective 1-carbon metabolism specifically involving folate and sarcosine ^31^. Pignataro et al. employed ribosome display approach to identify small molecules with may modulate FXN function. They found that treatment Affi_224 was able to increase cysteine NSF1 desulfurase activation in the context of specific FXN mutants ^32^. Other proteomics approaches have also been used to identify differences in cerebral spinal fluids of FRDA and control patients and several pathways associated with oxidation and inflammation were differentially regulated ^33^.

Our investigation endeavored to identify unique protein-protein interactions with FXN. We incorporated two complementary approaches, BioID (Figure 1) and a traditional proteomic approach, Co-IP (Figure 2) to identify protein interactions with FXN at the direct binding, indirect binding, and non-proximal levels. Using this integrated approach, our data indicated over 1000 proteins with significant affinity compared to the MTS-control. Using manual curation and verification from gold standard mitochondrial protein repositories, we chose to focus on the 41 novel protein interactions identified by both BioID and IP techniques. These 41 proteins were analyzed using proteins-protein interaction network (PPI), GeneMenia, using up to 6 levels of connections (Figure 3).

We incorporated FXN protein interaction network with FXN biological function using systems biology landscape, mitochondrial biology, and FRDA pathogenesis and focused on 20 selected biosystems and interaction with the 41 identified proteins. Using discovery workflow, 35 new direct and 18 indirect interactions were identified. Previous approaches have supported FXN-NFS1 interactions dependent on ISD11 ^28^ (Figure 4). Our analysis using both BioID or co-IP, did not detect ISD11 which reinforces the likelihood of a novel direct affinity between FXN and NFS1. This discovery may provide new insights into ISC assembly and function.

The 41 proteins identified in the FXN protein landscape were further analyzed using interaction type and functional pathways. We identified and chose peroxiredoxin 3 (Prdx3) as a specific target of interest because the FXN-Prdx3 pathway interactions and the relations to oxidative stress and mitochondrial organization are both pathologies associated with FRDA. PRDX3 is an oxidoreductase located primarily in the mitochondria. Its primary role is to remove peroxides from the mitochondrial environment. Prdx3 is transcriptionally regulated by the antioxidant transcription factor Nrf2. PRDX3 deficient mice have less mitochondrial DNA, increased mitochondrial injury, impaired ATP production, neuronal apoptosis, and decreased physical strength ^34,35^. Conversely, *PRDX3* overexpression prevents heart failure following myocardial infarction ^36^. Decreased Prdx3 expression was detected in dorsal root ganglia of YG8R mice that express mutant human frataxin ^16^, supporting an association between FXN and Prdx3 and overexpression of mitochondrial peroxiredoxin restored life span in a *Drosophila* model of FRDA ^37^. A recently approved therapeutic for FRDA, Omovaloxalone, is a Nrf2 inducer and likely induces expression of mitochondrial antioxidants including Prdx3.

This study has demonstrated the strengths of employing complementary methods to identify a unique interactome for FXN. Our data provides new insights into FXN function and regulation and a potential direct interaction between FXN and NFS1. We have also definitively identified an association between FXN and Prdx3 which has potential applications for mitochondrial pathology and future therapeutic strategies. As a weakness, the protein affinity approach does not capture the spatial representation involved in the BioID2 technology mechanism. Future work to integrate that knowledge in the algorithm will greatly improve the knowledge accuracy. However, this does not diminish our finding because our result relies more on the qualitative aspect of the protein interaction, and less on the quantification of the interaction.

## METHODS

### Cell culture.

Human lung adenocarcinoma A549 cells (CCL-185; American Type Culture Collection, Manassas VA) and HEK293 Phoenix cells (National Gene Vector Biorepository, Indianapolis IN) were cultured in 5% CO_2_ at 37°C in high glucose (25 mM) DMEM supplemented with 10% fetal bovine serum, 100 units/mL penicillin, and 100 μg/mL streptomycin (HyClone, Logan UT). The following lymphoblastoid cell lines from apparently healthy individuals were from obtained from the Coriell Cell Repository (Coriell Institute for Medical Research, Camden NJ): GM03798 (10 y male), GM05398 (44 y male), GM14406 (41 y female), GM14907 (28 y male), and GM07521 (19 y female). Lymphoblasts were cultured in 5% CO_2_ at 37°C in Gibco high glucose (25 mM) RPMI 1640 supplemented with 15% fetal bovine serum, 2 mM L-glutamine, 100 units/mL penicillin, and 100 μg/mL streptomycin (ThermoFisher Scientific, Waltham MA). Fibroblasts were obtained from the Coriell Institute for Medical Research or the Friedreich’s Ataxia Primary Fibroblast Repository^38^ maintained by David Lynch of The Children’s Hospital of Philadelphia and Marek Napierala of the University of Alabama at Birmingham. Fibroblasts were cultured in 5% CO_2_ at 37°C in DMEM/F12 media containing 10% FBS, 100 units/mL penicillin, 100 μg/mL streptomycin, and 1% non-essential amino acids (Life Technologies, Carlsbad CA).

### Plasmids & Generation of BioID2 cell lines.

FXN, PRDX3, and the Cox8A mitochondrial targeting sequence (MTS) were amplified by PCR from human cDNA and inserted into a previously generated BioID2-HA pBabe-puro vector (#74224; Addgene, Watertown MA)^39^ using EcoR1 and BamH1 (New England Biolabs, Ipswich MA) restriction sites following manufacturer’s instructions for the Takara Bio In-Fusion cloning method (Mountain View CA). Stable cells for all BioID2 constructs were generated using retroviral transduction. HEK293 Phoenix cells were transfected with FXN-BioID2, PRDX3-BioID2, and MTS-BioID2 constructs using Lipofectamine 3000 (ThermoFisher Scientific) for 6 h at 37°C, media was then replenished, and cells were moved to 32°C for a 72h incubation. Viral supernatant from transfected Phoenix cells was filtered (0.45 μm) then added to A549 target cells along with 0.5mL of fresh media and 0.25 μg/mL polybrene (Santa Cruz Biotechnology, Dallas TX). After 72 h after transduction, cells were plated into 10 cm dishes with fresh media and 2 μg/mL puromycin (ThermoFisher Scientific) was added for selection.

### SDS-PAGE & immunoblot.

As previously described^40^, cell lysates were diluted in Laemmli buffer, sonicated to shear DNA, and separated by polyacrylamide gel electrophoresis (Mini-PROTEAN TGX, Bio-Rad, Hercules, CA). To detect biotinylated proteins, gels were transferred to nitrocellulose membranes and probed with Streptavidin-HRP (1:5000, Abcam ab7403) diluted in 0.4% Triton X-100/PBS and imaged via enhanced chemiluminescent substrate detection on LiCor Odyssey FC (Lincoln NE). For detection with primary antibodies, membranes were blocked in Licor Odyssey blocking buffer (TBS) before incubating overnight at 4°C in chicken anti-BioID2 (1:5,000)^39^, mouse anti-NFS1 (1:500, MyBioSource MBS395145, San Diego CA) rabbit anti-FXN PAC 2571^27^, rabbit anti-mouse FXN (1:500, Abcam ab175402), rabbit anti-human FXN (1:500, Abclonal A1745, Woburn MA), rabbit anti-mouse/human Prdx3 (1:500, LF-PA0030 ThermoFisher Scientific), rabbit anti-mouse/human Prdx5 (1:500, AdipoGen Life Sciences LF-PA0210, San Diego CA), rabbit anti-mouse Trx2 (1:500, Abcam ab185544), rabbit anti-human Trx2 (1:500, Santa Cruz Biotechnology sc-20146), rabbit anti-mouse TrxR2 (1:500, Proteintech Group 16360–1-AP, Rosemont IL), rabbit anti-human TrxR2 (1:1,000, Abcam ab58445, Cambridge, United Kingdom), mouse anti-mouse β-actin (1:1,000, Abcam ab8226) or rabbit anti-human β-actin (1:1,000, Sigma Aldrich A2066,St Louis MO). Membranes were then incubated with IRDye680-conjugated anti-mouse (1:10,000 LiCor 925–68070) or anti-rabbit (1:10,000, LiCor 925–68071) and immune complexes were captured and analyzed on a LiCor Odyssey FC and Image Studio. Detection of rabbit anti-mouse FXN (Abcam) was achieved with HFP-conjugated anti-rabbit (1:5,000 SouthernBiotech 4030–05, Birmingham AL) and clarity max western ECL substrate (Bio-Rad 1705062).

### Immunocytochemistry.

Cells grown in fluorodishes were fixed with 3% wt/vol paraformaldehyde/phosphate-buffered saline (PBS) for 10 m and permeabilized by 0.4% Triton X-100 in PBS for 15 m. Cell were labeled with chicken anti-BioID2 (1:5,000)^39^ and 1:1,000 mouse anti-CoxIV (1:1,000, Abcam ab33985). Primary antibodies were detected using 1:1,000 Alexa Fluor 568-conjugated goat anti-chicken (1:1,000, ThermoFisher Scientific A11041) and Alexa Fluor 647-conjugated goat anti-mouse (1:1,000, ThermoFisher Scientific A11029) while Alexa Fluor 488-conjugated streptavidin (1:1,000, ThermoFisher Scientific S32354) was used to detect biotinylated proteins. DNA was visualized with Dapi. Confocal microscopy was performed using a Nikon A1 confocal microscope (60X/1.49 oil APO TIRF Nikon objective) with a charge-coupled CoolSnap HQ camera (Photometrics) linked to a workstation running NIS-Elements software (Nikon, Melville NY).

### Affinity capture of biotinylated proteins.

All BioID pulldowns were performed in triplicate as described in detail^41^. For each BioID2-fusion line, 2 × 10^7^ cells were incubated with 50 μM biotin for 16–18 h. Cells were washed twice with PBS and lysed in 1.08 mL of lysis buffer containing 8 M urea, 50 mM Tris pH 7.4, 1 mM DTT, and 1X Halt protease inhibitor (ThermoFisher Scientific). Triton X-100 was added to final concentration of 1% and cells were sonicated twice on ice to shear DNA (45 s at 30% duty cycle and output level 3, Branson Sonifier-250). Detergent was diluted by adding an equal volume of lysis buffer, and sonication was repeated once more. Lysates were centrifuged at 16,5000 × *g* for 10 m, and supernatants were collected to a 5 mL Eppendorf tube and precleared with 200 μL gelatin Sepharose 4B beads (GE Life Sciences, Pittsburgh PA) rotating for 2 h at 4°C. Samples were then centrifuged at 800 × *g* for 5 m and supernatant transferred to a fresh 5 mL Eppendorf tube. Affinity capture was performed by adding 50 μL streptavidin Sepharose high performance beads (GE Life Sciences) and incubating on rotator for 4 h at 4°C. Beads were collected by centrifugation at 800 × *g* for 5 m, and washed four times with wash buffer (8 M urea in 50 mM Tris pH 7.4). Beads were resuspended in 1 mL wash buffer and 100 μL was taken for SDS-PAGE & immunoblot while the other 90% of sample was resuspended in 50 mM NH_4_HCO_3_ supplemented with 1 mM biotin for LC-MS/MS analysis.

### BioID on-bead protein digestion and identification by 1D LC-MS/MS.

Beads were thawed and resuspended with 8 M urea and 50 mM ammonium bicarbonate and cysteine disulfide bonds were reduced with 10 mM tris(2-carboxyethyl)phosphine (TCEP) at 30°C for 60 m followed by cysteine alkylation with 30 mM iodoacetamide in the dark at room temperature for 30 m. Following alkylation, urea was diluted to 1 M urea using 50 mM ammonium bicarbonate, and proteins were subjected to overnight digestion with mass spec grade Trypsin/Lys-C mix (Promega, Madison WI). Beads were pulled down, supernatant with peptides was collected, and beads were washed with 50 mM ammonium bicarbonate to increase peptide recovery. Following digestion, samples were acidified with formic acid (FA) and subsequently desalted using AssayMap C18 cartridges mounted on an Agilent AssayMap BRAVO liquid handling system, C18 cartridges were first conditioned with 100% acetonitrile (ACN), followed 0.1% FA. Sample was then loaded onto the conditioned C18 cartridge, washed with 0.1% FA, and eluted with 60% ACN, 0.1% FA. The organic solvent was removed in a SpeedVac concentrator prior to LC-MS/MS analysis. Before injecting the sample in the LC-MS, total sample peptide amount was determined by NanoDrop spectrophometer (ThermoFisher Scientific). Dried samples were reconstituted with 2% acetonitrile, 0.1% formic acid and analyzed by LC-MS/MS using a Proxeon EASY nanoLC system coupled to an Orbitrap Elite mass spectrometer (ThermoFisher Scientific). Peptides were separated using an analytical C18 Acclaim PepMap column 0.075 × 500 mm, 2 μm particles (ThermoFisher Scientific) in a 120-min gradient of 2–28% solvent B at a flow rate of 300 nL/min. The mass spectrometer was operated in positive data-dependent acquisition mode. MS1 spectra were measured with a resolution of 60,000, an AGC target of 1 × 10^6^ and a mass range from 350 to 1400 m/z. Up to 10 MS2 spectra per duty cycle were triggered, fragmented by collisium-induced dissociation, and acquired in the ion trap with an AGC target of 1 × 10^4^, an isolation window of 2.0 m/z and a normalized collision energy of 35. Dynamic exclusion was enabled with duration of 30 s. All mass spectra from were analyzed with MaxQuant software (v1.5.5.1).

### Frataxin co-immunoprecipitation and identification by 1D LC-MS/MS.

Lymphoblastoid cell lysates were separated by Superdex 75 size exclusion chromatography. High molecule weight fractions were pooled and immunoprecipitation was performed with rabbit anti-FXN PAC 2517 immobilized on Protein A Magnetic beads, as described^27^. Aliquots of pooled sample before immunoprecipitation (input, ~5% of total volume), the flow-through fraction (not bound; ~5% of total volume), and the affinity-purified fraction (bound; 100% of total volume) were analyzed by SDS-PAGE & immunoblot and bound lysate was digested for LC-MS/MS.

### FXN-protein interaction framework.

A computational workflow followed by manual curation was used for proteomic discovery of FXN protein networks with biological relevance within the context of FXN function and FRDA pathogenesis. The workflow (1) assessed the quality of the raw data, (2) determined statistical significance, (3) validated existing physical interactions with FXN, (4) predicted potential new physical interactions with FXN, and (5) assembled a functional annotation to identify molecular ecosystems represented within the FXN-protein network.

#### Determination of novel FXN-protein interactions.

GENEMANIA and STRINGdb databases were used to identify new FXN-protein interactions and determine the level of the interaction. Proteins directly downstream from FXN were labeled as level 1 (L1), the second protein interacting with FXN were labeled level 2 (L2), and so on. Following the concept underlying BioID2 technology, we considered L1 proteins more likely to directly interact (Pd) with FXN than and considered protein interactions at L2 and L3 as indirect interactions (Pi) (Fig. 3D). Analysis of our interaction collection (supplemental file 3) lead to identification of 35 new direct interactions and 18 new indirect interactions with FXN (Fig. 3E, Table 1).

### Bioinformatics.

The computational workflow used descriptive statistics and boxplot to check data distribution and outliers, followed with a linear normalization to allow significance analysis between BioID2 target and MTS control affinity profiles using ProteoSign^42^. SAINTexpress^43^ was used to assess statistical probability of candidate contaminants. Manual curation of proteins with less than two spectral counts out of the three replicates were removed due to the lack of confidence. Proteins identified from MTS-BioID2 cells were used to remove false positive candidates from FXN-BioID2 and Prdx3-BioID2 samples. However, proteins whose label free quantification values were threefold more than those in MTS-BioID2 controls were also considered candidates. Proteins had to be identified on at least one of the following mitochondrial localization reference databases: MitoCarta2.0, Integrated Mitochondrial Protein Index (IMPI), and mitochondrion gene ontology. Candidate protein/gene functional interaction networks, pathways, and localizations were analyzed using GeneMania^44^ and Networkanalyst.ca^45^. An integrated, comprehensive network analysis was done using PSIQUIC^46^ then results were integrated with Cytoscape3^47^ to determine shortest-path networks. Finally, a level-based network analysis of direct and indirect protein-protein interactions was used to identify network hubs and map the type of network interaction as well as any known or new molecular functions. Mass spectrometry proteomics data were deposited to the ProteomeXchange Consortium via the PRIDE^48–50^ partner repository (dataset identifier PXD015034).

### Prdx3 redox status.

Cells were washed with ice-cold PBS then lysed in alkylation buffer containing 100 mM N-ethylmaleimide (NEM) and 10 μg/mL catalase to label and retain reduced monomers for non-reducing SDS-PAGE/immunoblot analysis^51^.

### Approvals for studies with experimental animals.

All animal experiments were conducted with approval from the Sanford Research Institutional Animal Care and Use Committee (protocol number 142-03-21E). Sanford Research has an Animal Welfare Assurance on file with the Office of Laboratory Animal Welfare (A-4568-01) and is a licensed research facility under the authority of the United Sates Department of Agriculture (46-R-0011).

### Mouse strains, care & behavior testing.

All animal experiments were conducted with approval from the Sanford Research Institutional Animal Care and Use Committee (protocol number 142-03-21E). All methods were carried out in accordance with relevant guidelines and regulations. Sanford Research has an Animal Welfare Assurance on file with the Office of Laboratory Animal Welfare (A-4568-01) and is a licensed research facility under the authority of the United Sates Department of Agriculture (46-R-0011). All methods are reported in accordance with ARRIVE guidelines (https://arriveguidelines.org). FRDA knockdown (FRDAkd) mice obtained from Daniel Geschwind (University of California, Los Angeles) have doxycycline permissible expression of shRNA targeting *Fxn* ^30^. Mice were provided food and acidified water ad libitum and monitored daily. Genomic DNA was prepared using the AccuStart II Mouse Genotyping Kit (Quanta Biosciences, Gaithersburg MD) and genotyping were performed using two primer sets for detection of FRDAkd and control Igβ: FRDAkd 5’ CCATGGAATTCGAACGCTGACGTC; FRDAkd 3’ TATGGGCTATGAACTAATGACCC; Igβ 5’ GAGACTCTGGCTACTCATCC; Igβ 3’ CCTTCAGCAAGAGCTGGGGAC. FXN shRNA expression was induced at 10–14 weeks-old using 2 mg/mL doxycycline hyclate (Caymen Chemical 14422, Ann Arbor MI) in drinking water supplemented with 5% sucrose for 18 weeks. Animal subjects were euthanized by asphyxiation followed by cervical dislocation. Ataxic scoring summarized by Guyenet et al. was comprised of hind limb clasping, the ledge test, and gait analysis. A grip strength meter was used to measure all-limb grip strength (Columbus Instruments, Columbus OH). As a mouse grasped the meshing with forelimbs and hindlimbs, the peak pull force in grams was recorded on a digital force transducer.

### Statistics.

Values represent mean ± standard deviation of biological replicates. Group means were compared by 1-way ANOVA using Bonferroni’s post hoc test while student’s *t* test was used for comparison between means of two sets of data with GraphPad Prism 8 (GraphPad Software, San Diego CA). Outliers were identified utilizing the ROUT method (Q=1%) and statistical significance was defined as p≤0.05.

## Data Availability

Project Name: Discovery of frataxin mitochondrial networks reveals key regulators of Friedreich’s Ataxia Project accession: PXD015034 Project DOI: Not applicable Reviewer account details: Website: http://www.ebi.ac.uk/pride Username: reviewer56541@ebi.ac.uk Password: twBbqVfM Data are available via ProteomeXchange with identifier PXD015034

## Abbreviations

FRDA: Friedreich’s Ataxia
FXN: frataxin
ISC: iron-sulfur cluster
Nrf2: nuclear factor erythroid 2-related factor 2
COXIV: cytochrome oxidase subunit IV
Prdx3: peroxiredoxin 3
Prdx5: peroxiredoxin 5
Trx2: thioredoxin 2 and thioredoxin reductase 2 (TrxR2), between disease and control states
FRDAkd: FRDA knockdown
